# Reduced selection leads to accelerated gene loss in *Shigella*

**DOI:** 10.1186/gb-2007-8-8-r164

**Published:** 2007-08-08

**Authors:** Ruth Hershberg, Hua Tang, Dmitri A Petrov

**Affiliations:** 1 Department of Biological Sciences, Stanford University, Serra Mall, Stanford, CA 94305, USA; 2 Department of Genetics, Stanford University, Serra Mall, Stanford, CA 94305, USA

## Abstract

**Background:**

Obligate pathogenic bacteria lose more genes relative to facultative pathogens, which, in turn, lose more genes than free-living bacteria. It was suggested that the increased gene loss in obligate pathogens may be due to a reduction in the effectiveness of purifying selection. Less attention has been given to the causes of increased gene loss in facultative pathogens.

**Results:**

We examined in detail the rate of gene loss in two groups of facultative pathogenic bacteria: pathogenic *Escherichia coli*, and *Shigella*. We show that *Shigella *strains are losing genes at an accelerated rate relative to pathogenic *E. coli*. We demonstrate that a genome-wide reduction in the effectiveness of selection contributes to the observed increase in the rate of gene loss in *Shigella*.

**Conclusion:**

When compared with their closely related pathogenic *E. coli *relatives, the more niche-limited *Shigella *strains appear to be losing genes at a significantly accelerated rate. A genome-wide reduction in the effectiveness of purifying selection plays a role in creating this observed difference. Our results demonstrate that differences in the effectiveness of selection contribute to differences in rate of gene loss in facultative pathogenic bacteria. We discuss how the lifestyle and pathogenicity of *Shigella *may alter the effectiveness of selection, thus influencing the rate of gene loss.

## Abstract 2

The rate of gene loss was studied in the facultative pathogens, *E. coli *and *Shigella*, and was found to be greater in the more niche-limited Shigella. This is demonstrated to be due to a genome-wide reduction in the effectiveness of selection.

## Background

It was long thought that mutations in the sequences of individual genes are the strongest contributors to evolutionary change. In recent years, evidence has accumulated showing that the emergence of new strains of pathogenic bacteria can be better explained by changes in the repertoire of genes through gene acquisition and gene loss [1-3]. Obligate pathogens tend to lose a very high number of genes compared with facultative pathogens, which, in turn, harbor a larger number of pseudogenes than free-living bacteria [3]. It was postulated that the observed increase in gene loss in obligate pathogens is due to two types of reduction in purifying selection [2,4-6], pathway-specific reduction and genome-wide reduction. In pathway-specific reduction, specific functions that are carried out by free-living bacteria may be provided to a certain extent by the host of the pathogenic bacteria, or may not be needed once a pathogen adapts to survival within a host. For this reason, purifying selection may be less effective in preventing the loss of some genes involved in specific pathways that are no longer as useful as they were in the free-living ancestor of the pathogenic bacteria. In genome-wide reduction, population size and structure may be different in pathogens compared with free-living bacteria. Specifically, population size is likely to be reduced in obligate pathogens. These differences may influence the effectiveness of selection. For this reason, all genes, independent of the pathways in which they are involved, may be more readily lost in pathogens than in free-living organisms. While these two sources of reduction in the effectiveness of selection were previously described regarding obligate pathogens, it is reasonable that they may also play a role in determining the rate of gene loss in facultative pathogens.

In addition to changes in the efficacy of purifying selection, changes in the patterns of positive selection may also play a role in determining gene loss. Specifically, the products of some genes may be detrimental to pathogenicity and their loss from a pathogen's genome may be adaptive [1]. For instance, in *Shigella*, the loss of the *cadA *gene, encoding the lysine decarboxylase, was shown to correlate with an increase in pathogenicity [7-9]. Also, genes that encode cell-surface determinants are sometimes adaptively lost from pathogen genomes [1], presumably in order to avoid the restrictive effects of the host immune response. This has been observed in pathogens such as *Shigella *[10], *Bordetella *[11], and *Mycobacterium tuberculosis *[12].

In this study we examined the relationship between gene loss and the effectiveness of selection in 12 fully sequenced facultative pathogenic *Escherichia coli *and *Shigella *strains. Different strains of pathogenic *E. coli *may infect a variety of hosts and cause a number of intestinal as well as extra-intestinal diseases [13]. In contrast, all *Shigella *strains infect only humans and closely related primates. They invade the cells of infected individuals and cause a specific disease (Shigellosis, or bacillary dysentery) [7,14,15]. Historically, *Shigella *and *E. coli *have been classified as two distinct species. However, more recent studies indicate that *Shigella *strains have been derived repeatedly from different branches of the *E. coli *strain tree through independent acquisition of the pINV virulence plasmid [7,13-15].

Here, we examine gene loss along the branches of the *E. coli*/*Shigella *strain tree and demonstrate a significantly accelerated rate of gene loss along the branches leading towards the *Shigella *strains. We demonstrate that at least some of the variation observed in the rate of gene loss can be explained by a genome-wide reduction in the effectiveness of purifying selection along the *Shigella *branches of the tree.

## Results

### More *E. coli *K12 genes are absent from *Shigella *than from other pathogenic *E. coli *strains

We compared the protein-coding gene repertoire of the well-annotated non-pathogenic lab strain *E. coli *K12 to that of the six fully sequenced pathogenic *E. coli *strains and the six fully sequenced *Shigella *strains. We examined whether each of the 4,183 protein-coding genes of *E. coli *K12 is present or absent in each of the studied *E. coli *and *Shigella *strains. In order to focus on gene loss, we eliminated from our examination genes that are predicted to be horizontally transferred into *E. coli *K12 [16]. We also wanted to minimize the number of cases in which a gene is falsely annotated in *E. coli *K12. To this end, we removed from consideration all genes that have no functional annotation in the *E. coli *annotation database, Ecogene [17]. This left us with a dataset of 2,394 genes. It is immediately apparent that more *E. coli *K12 genes are absent from *Shigella *strains than from *E. coli *strains (Table 1).

**Table 1:** Number of *E. coli *K12 genes absent from the studied pathogenic *E. coli *and *Shigella *strains

Strain	No. of *E. coli *K12 genes absent from strain
*E. coli *O157:H7	132
*E. coli *O157:H7 EDL933	135
*E. coli *APEC 01	148
*E. coli *UTI89	138
*E. coli *CFT073	174
*E. coli *536	180
*Shigella sonnei *Ss046	255
*Shigella flexneri *2a	371
*Shigella flexneri *2a 2457T	353
*Shigella flexneri *5 8401	347
*Shigella boydii *Sb227	366
*Shigella dysenteriae *Sd197	543

### Gene loss along branches of the *E. coli *and *Shigella *phylogenetic tree

**Figure 1:**
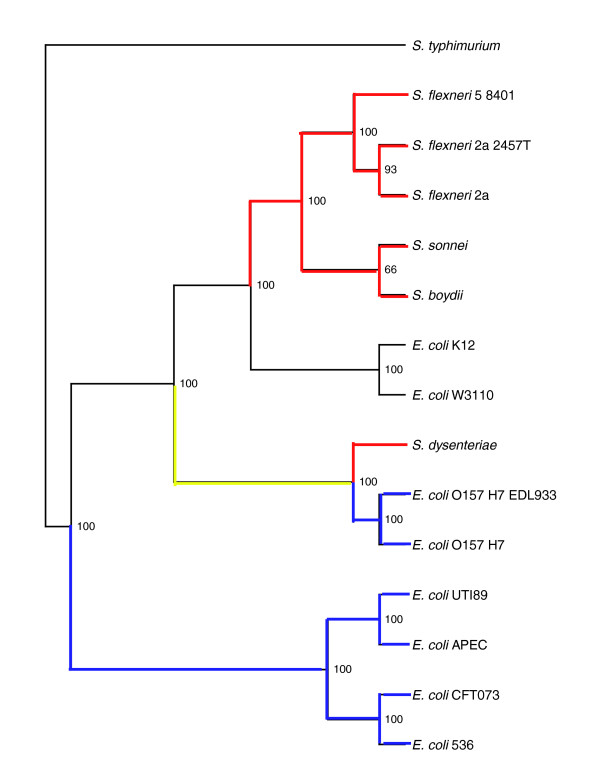
Phylogenetic tree representing *E. coli *and *Shigella *strains. The tree was built based on an alignment of the concatenated sequences of 100 genes that were selected at random from a list of 1,214 *E. coli *K12 genes that are present and can be fully aligned in all the pathogenic *E. coli *strains, the *Shigella *strains and in *S. typhimurium*. Bootstrap analysis was carried out with 100 replicas. The number of replicas that agree with each branch assignment are indicated. Branches of the tree that lead towards a pathogenic *E. coli *strain are colored blue. Branches of the tree that lead towards a *Shigella *strain are colored red. If a node in the tree is a direct ancestor only of pathogenic *E. coli *strains, it was considered to be a pathogenic *E. coli *strain itself. Similarly, if a node is a direct ancestor only of *Shigella *strains it is considered to be a *Shigella*. Under these assumptions, it is not clear whether the direct ancestor of the EHEC strains and of *S. dysenteriae *should be classified as a pathogenic *E. coli *or rather as a *Shigella*. The branch connecting this group to its ancestor is colored yellow.

A phylogenetic tree was constructed for the organisms studied, using *Salmonella typhimurium *LT2 as an outgroup. The tree was constructed based on the alignment of the concatenated sequences of 100 genes that are conserved in all the examined strains and that were selected at random (Materials and methods). In order to further confirm the reliability of the tree, we applied bootstrap analysis using 100 replicas (Materials and methods). The resulting tree is shown in Figure 1. Constructing phylogenies for bacterial strains is complicated by the frequent occurrence of horizontal gene transfer. We sought to minimize this problem by excluding from our analysis those sequences that were predicted to be horizontally transferred into *E. coli *K12. In addition, we used a large number of concatenated gene sequences to build our tree. Even if some of these genes were horizontally transferred into some of the strains studied, we believe that this noise should be diluted within our dataset. As can be seen in Figure 1, most branches of the tree have very high bootstrap support.

Since we removed from consideration those genes predicted to have been horizontally transferred into *E. coli *K12, we assume that for the remaining genes horizontal gene transfer is negligible. We can thus postulate that if two bacteria, *a *and *b*, share an immediate ancestor, *A*_*a*.*b*_, and a gene is absent from *a *but present in *b*, it was present in *A*_*a*.*b *_and was lost on the branch that connects *A*_*a*.*b *_to *a*. Based on this we assessed the most likely branches along which each gene was lost and calculated the number of *E. coli *K12 genes lost along each branch of the tree. Genes that were duplicated in *E. coli *K12 after it split from the other strains may be falsely annotated by this scheme as having been lost in all of the other strains examined. As the organisms examined are very close, we do not expect to see many such cases. In addition, since such genes will be counted as lost in all branches, they should not affect our analysis, which is comparative by nature.

Of the *E. coli *K12 genes studied, 1,214 were found to be conserved in all the *E. coli *and *Shigella *strains considered and in *S. typhimurium*. We aligned the sequences from all of these 1,214 genes separately. We ran the PAML Codeml program [18] on these alignments using the 'all branch', free-ratio model in order to estimate the rate of synonymous (dS) and non-synonymous (dN) substitutions per site along each branch of the tree. Average dS and dN values were then calculated for each branch of the tree. While single gene estimates of dS and dN may be rather noisy, we averaged these values across a large number of genes and, therefore, believe that the average estimates we received are informative. Generally, the average dS can be taken as a measure of the age of the branch, while the average dN measures both time and the genome-wide effectiveness of selection along a branch. As the strains studied are very closely related, the dS values we calculated are small. This indicates that saturation of dS values is not a problem in this dataset.

**Figure 2:**
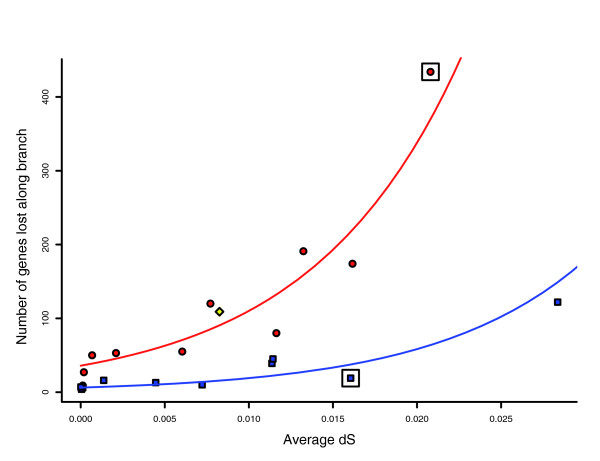
Genes are lost at an accelerated rate along branches that lead towards *Shigella strains*. Plotted is the number of genes lost along the branches of the phylogenetic tree against the average dS along the same branches. Red circles represent branches that lead towards *Shigella *strains. Blue squares represent branches that lead towards pathogenic *E. coli *strains. The red line represents the fitted Poisson model for the *Shigella *branches. The blue line represents the same for the pathogenic *E. coli *branches. The yellow diamond represents the branch that leads towards the direct ancestor of the EHEC strains and of *S. dysenteriae*. The blue square that is enclosed by a rectangle represents the branch that leads from the ancestor of the EHEC strains and *S. dysenteriae *to the ancestor of the two EHEC strains. The red circle that is enclosed by a rectangle represents the branch leading from the same ancestor towards *S. dysneteriae*.

It is reasonable to assume that the number of genes lost increases over time. Since dS approximates evolutionary time, it is not surprising that gene loss is positively correlated with the average dS along a branch (*r*_*spearman *_= 0.79, *P *≤ 3.41e-5). However, differences in the age of the *Shigella *and *E. coli *branches do not seem to fully explain the higher number of genes lost along the *Shigella *branches. When the number of genes lost along each branch is plotted against the average dS (Figure 2) it appears that gene loss tends to occur at an accelerated rate along the branches leading towards *Shigella *strains (red) compared with those leading toward *E. coli *strains (blue). To test formally whether both dS and the type of branch (*Shigella *versus *E. coli*) influence the rate of gene loss, we used a Poisson regression model (see Materials and methods). We modeled the number of genes lost along a branch as a Poisson random variable, whose mean parameter may depend on dS and on a binary strain indicator variable that receives the value one if the branch leads towards a *Shigella *strain and the value zero if it leads towards a pathogenic *E. coli *strain. We found that both dS and the strain indicator variable are statistically significant (*P *< 2 × 10^-16 ^for both variables). Furthermore, the regression coefficient corresponding to the strain indicator variable is positive ($\hat{\beta }$ = 1.76), confirming our hypothesis that, for a given dS value, genes are lost at an accelerated rate along the *Shigella *branches compared to the *E. coli *branches.

**Figure 3:**
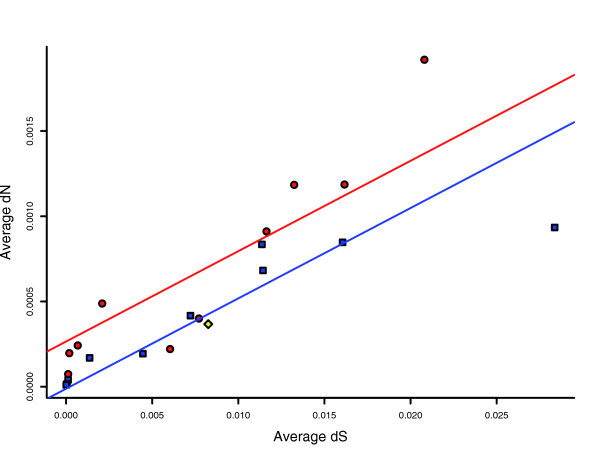
Reduced genome-wide effectiveness in selection along the *Shigella *branches. The average dN is plotted against the average dS. Red circles represent branches that lead towards *Shigella *strains. Blue squares represent branches that lead towards pathogenic *E. coli *strains. The red line represents the fitted linear model for the *Shigella *branches. The blue line represents the same for the pathogenic *E. coli *branches. The yellow diamond represents the branch that leads towards the direct ancestor of the EHEC strains and of *S. dysenteriae*.

It is possible that the accelerated rate of gene loss along the *Shigella *branches is due to a reduction in the effectiveness of selection. In order to examine whether there is, indeed, a reduction in the effectiveness of selection along the *Shigella *branches, we plotted the average dN against the average dS of each branch (Figure 3). If the effectiveness of selection is reduced for the *Shigella *strains, we expect higher dN values along a *Shigella *branch than along an *E. coli *branch for similar dS values. Indeed, a linear regression of dN on dS and the branch type indicates that, for similar values of dS, dN values tend to be higher along the *Shigella *branches (*P *= 0.0217).

The average dN along each branch correlates with gene loss even more strongly than does the average dS (r_*spearman *_= 0.82, *P *= 5.52e-6). Using Poisson regression models (see Materials and methods) we show that dN adds information about the rate of gene loss beyond that implicit in dS (LR = 620, df = 1, *P *< 2 × 10^-16^). It thus appears that a reduced genome-wide efficiency in selection may explain at least part of the acceleration observed in the rate of gene loss in *Shigella*.

When analyzing the relationship between dS and the numbers of genes lost, we did not take codon-bias into account. While dS should be less affected than dN by the strength of purifying selection, for some codon-biased genes, synonymous mutations may also be under purifying selection. For this reason, the average dS should be somewhat lowered by purifying selection. As selection is weaker along the branches leading towards the *Shigella *strains, dS should be less affected by codon-bias, along the *Shigella *branches when compared to the *E. coli *branches. Note that this is conservative for our purposes, given that this would mean that for a similar amount of time the average dS should be higher in the *Shigella *strains compared to the pathogenic *E. coli *strains. Thus, we are likely to overestimate the age of the *Shigella *branches and underestimate the rate of gene loss in the *Shigella *strains relative to that in *E. coli*. The true acceleration of gene loss in *Shigella *is thus likely to be even higher.

### Genes under less constraint are lost more readily

Our results indicate that the genome-wide effectiveness of purifying selection plays a role in determining the rate at which genes will be lost. If this is indeed the case and a reduction in selection occurs across the genome independently of the function of genes, it is likely that the genes that will be most readily lost are those that have been under weaker purifying selection.

In order to test this expectation, we calculated a pairwise dN/dS for each gene in each of the *E. coli *and *Shigella *strains against the reference sequence of the same gene from *E. coli *K12. These dN/dS values were then normalized within each strain by calculating a Z-score (see Materials and methods) and an average was calculated for all the strains in which the gene is present. Next, we divided the genes based on the number of times they appear to have been lost along the *E. coli*/*Shigella *strain tree (Table 2). In order to examine whether genes that are lost more often have higher dN/dS values in the strains in which they are conserved, we conducted one-tailed Mann-Whitney tests to compare each group to the group that precedes it. For instance, we compared whether genes that are lost once evolve significantly faster than genes that are never lost. The average dN/dS of each group is higher than that of the group that precedes it (Table 2) and this difference is statistically significant (*P *< 0.05) for all of the comparisons (Table 2).

**Table 2:** Genes under lower evolutionary constraint are lost more readily

No. of times lost along the *E. coli*/*Shigella *phylogenetic tree	No. of genes*	Average normalized dN/dS	*P *value^†^
0	1,510	-0.1704	NA
1	403	0.0278	1e-14
2	252	0.0967	0.0454
3	125	0.2041	0.0223
4-5^‡^	33	0.8347	0.0144

*A normalized dN/dS could not be calculated for 73 out of the 2,394 genes that were used in this study. Of these 73 genes, 45 are absent from all of the pathogenic strains studied and the additional 28 could not be fully aligned to the *E. coli *K12 sequence for any of the studied strains. For this reason the total number of genes summarized in this table is 2,321. ^†^Genes were divided based on the number of times they are lost and each group was compared to the preceding group. The *P *value shown is the *P *value with which the following null hypothesis can be rejected: the genes in this group have average dN/dS that are smaller or equal to the dN/dS values of the preceding group. ^‡^Genes lost four or five times were grouped together due to their small numbers

### Shifts in rates of gene loss may reflect shifts in pathogenicity and lifestyle

One of the branches in the examined pathogenic *E. coli*/*Shigella *strain tree could not be easily classified as an *E. coli *or *Shigella *branch. This is the branch that leads towards the ancestor of the two enterohaemorrhagic *E. coli *(EHEC) strains (*E. coli *O157:H7 and *E. coli *O157:H7 EDL933) and the *Shigella dysenteriae *strain. It is unclear whether this ancestor was a *Shigella*, an *E. coli*, or something in between. However, it is interesting to note that genes are lost along this branch at a rate comparable to that of the *Shigella *branches (Figure 2). The rate of gene loss observed along the branch leading from this ancestor of EHEC and *Shigella dyseneteriae *towards the two EHEC strains fits with the slower rate observed for *E. coli *branches (Figure 2). In contrast, the rate along the branch leading from the same ancestor to *S. dysenteriae *is more fitting with that observed for other *Shigella *branches (Figure 2). It is tempting to speculate that the ancestor of the EHEC strains and of *S. dysenteriae *was heading on the evolutionary path toward becoming a *Shigella*. It is possible that one of its descendants then continued on the *Shigella *pathway and became *S. dysenteriae *while another lost the *Shigella *invasion plasmid and reverted back to a lower rate of gene loss, which led to the evolution of the EHEC strains.

**Figure 4:**
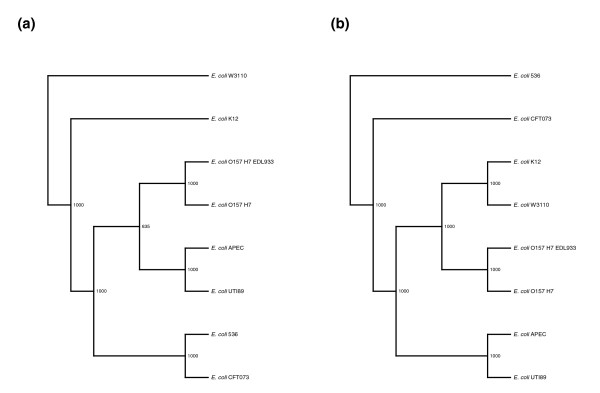
The *E. coli *strain phylogeny inferred based on **(a) **the *prpB *gene sequences is different from the more reliable phylogeny inferred based on **(b) **the concatenated sequences of 100 randomly selected genes. For each of the two trees, bootstrap analysis was applied using 1,000 replicas. The number of replicas that agree with each branch assignment is indicated. Unlike in the 'true' tree represented in Figure 1 and in (b), the *prpB*-tree shows the EHEC strains, *E. coli *O157:H7 and *E. coli *O157:H7 EDL933 to be closer to *E. coli *UTI89 and *E. coli *APEC 01 than to the lab strains of *E. coli*. This may indicate that the *prpB *was horizontally transferred into the EHEC strains.

Fitting with this speculation, we found an anecdotal indication that the *prpB *gene, which encodes a phosphoprotein phosphatase and has been implicated in the confinement of *Shigella *to their limited niche [19], may have been lost in the branch leading towards the EHEC-*S. dysenteriae *ancestor and was then regained in the EHEC strains through horizontal gene transfer. Namely, when we constructed a phylogenetic tree of the *E. coli *strains based on the *prpB *sequence (Figure 4a) we obtained a different tree than the consensus tree that we obtain by analyzing the alignment of the concatenated sequences of 100 genes that were selected at random (Figure 4b). While the difference in the maximum likelihood scores of the two trees, given the *prpB *sequence data is at best marginally statistically significant (*P *= 0.082 by the Shimodaira-Hasegawa test [20]), it may still be of interest. The tree in Figure 4b is more likely to represent the true phylogeny of the *E. coli *strains. Thus, if the *prpB *sequence gives a different tree, in which the EHEC strains are closer to *E. coli *UTI89 and *E. coli *APEC 01 than to the lab strains of *E. coli*, it may indicate that *prpB *has been horizontally transferred.

## Discussion

It has been suggested in the past that obligate pathogens lose more genes than free-living organisms, due to differences in the effectiveness of purifying selection [2-4]. Here we focus on gene loss in facultative pathogenic *E. coli *and *Shigella *strains. While *Shigella *are considered to be clones of *E. coli *and have been derived repeatedly from different branches of the *E. coli *tree [7,13-15], we show that they are losing genes at an accelerated rate compared to other pathogenic *E. coli *strains. We demonstrate that this accelerated rate of gene loss can be partially explained by a genome-wide reduction in the effectiveness of purifying selection on the branches leading towards the *Shigella *strains. This genome-wide reduction in purifying selection may be explained by differences in the population structure and population size of *Shigella *compared with the other pathogenic *E. coli *strains. *Shigella*'s only natural hosts are humans, although it can infect other higher primates as well [7]. In addition, it has been shown that as little as 200 *Shigella *cells are sufficient to cause dysentery [13]. It is possible that its limited host-range together with the relatively small dosage needed to cause infection lead to a reduction in the effective population size of *Shigella *that, in turn, leads to the observed reduction in the effectiveness of selection

Our analysis demonstrates that differences in the genome-wide effectiveness of purifying selection contribute significantly to the accelerated rate of gene loss in *Shigella*. In addition, it is possible that some of the acceleration in the rate of gene loss is attributable to a higher incidence of pathway-specific reduction in purifying selection. *Shigella *are intracellular pathogens and as such occupy a very different niche than most *E. coli *strains. This lifestyle may explain why *Shigella *has no need for several of the pathways that are known to be lost in several *Shigella *strains [7,13]. For instance, *Shigella *does not biosynthesize flagella, due to its ability to utilize actin-assisted motility in order to move intracellularly [7,21]. In addition, *Shigella *is more host-specific than the other strains considered [7,13]. It appears that compared to the other *E. coli *strains studied, *Shigella *may be more limited in the niche it can occupy, which may explain why more pathways can be readily lost in *Shigella*.

It thus appears that the increased rate of gene loss in *Shigella *may be the result of its increased host specificity. Previous studies have also shown a link between host-specificity and higher incidence gene loss: Normand *et al*. [22] studied strains of the facultative plant symbiont *Frankia *and showed that genome size in these bacteria reduces with an increase in host specificity. An association between gene loss and increase in host specificity was also demonstrated for facultatively pathogenic strains of *Bordetella *and *Salmonella *[11,23]. Unlike other serovars of *Salmonella*, *Salmonella typhi *and *Salmonella paratyphi A *are limited to infecting only humans. Both serovars have a similar pahtogenicity phenotype [23]. Interestingly, it was demonstrated that while both of these *Salmonella *serovars accumulated a large amount of pseudogenes, the number of genes that have been commonly lost in both serovars is very small [23]. This may support our finding that following a reduction in host-range much of the increase in gene loss is due to a reduction in the effectiveness of purifying selection that is genome-wide rather than pathway specific.

On the one hand, high niche specificity may lead to both genome-wide and pathway specific reductions in the effectiveness of purifying selection, which, in turn, may lead to accelerated gene loss. On the other hand, accelerated gene loss may increase niche specificity as more and more functions are removed from the genome. This raises an interesting chicken-egg type question: is reduction in the selection against gene loss the result of the increased niche specificity of *Shigella*, or is it its cause? It is possible that both are true and that this is an autocatalytic process. After acquiring the invasion plasmid that allows *Shigella *strains to invade mucosal epithelium cells, some genes may be lost. This in turn limits the possibility of a *Shigella *strain to renege on this strategy and forces it to remain in the limited niche it now occupies. Once *Shigella *occupies this limited niche, reduction in purifying selection leads to an additional loss of genes at an accelerated rate.

At the early stages of the process it may be possible for an evolving *Shigella *strain to lose its invasion plasmid, gain some genes back through horizontal gene transfer and revert back to the *E. coli *phenotype. We show a possible example of such a case. It appears that the ancestor of the two EHEC strains and the *S. dysenteriae *strain was losing genes at a rate comparable to that of *Shigella*. It is possible that this ancestral strain carried the invasion plasmid and was starting on the evolutionary path to becoming a *Shigella*. While some of its descendants may have continued to lose genes at an accelerated rate, resulting in the *S. dysenteriae *strain we see today, others may have reverted back to the *E. coli *rate of gene loss leading to the evolution of the EHEC strains. In the case described above, the ancestor of EHEC and *S. dysneteriae *may have lost the *prpB *gene that seems to have been re-gained by horizontal gene transfer in the ancestor of the two EHEC strains. The *prpB *gene encodes a phosphoprotein phosphatase that functions in signal transduction pathways for the degradation of misfolded proteins [19]. A recent study [19] has shown that *prpB *is extremely susceptible to loss in *Shigella *strains. Of 58 strains examined, only 12 encoded an intact *prpB *open reading frame. It was suggested that losing the prp function limits the tolerance of the *Shigella *strains to external stresses. This in turn may reduce the environmental elasticity of *Shigella *and thus further limit the niche width for these bacteria. While *prpB *is absent from 46 of 58 strains examined by Li *et al*. [19] and is also absent from the 6 fully sequenced *Shigella *strains used in this study, it is present in all fully sequenced *E. coli *strains. It thus appears that in order to revert back to the *E. coli *lifestyle, the ancestor of the EHEC strains may have had to regain the prp function through horizontal gene transfer.

It would be interesting to learn whether there is a point of no return after which a *Shigella *strain has lost so many genes that it would no longer be able to regain enough functions to escape niche limitation. If such a point of no return exists, then the *Shigella *phenotype may represent an evolutionary dead-end. While *Shigella *species evolve repeatedly from different strains of *E. coli*, it might be much harder for a *Shigella *strain to give rise to a strain of *E. coli*.

Some cases of gene loss may be adaptive. For example, it has been shown that lysine decarboxylation is detrimental to *Shigella *pathogenicity and that the introduction of the *cadA *gene encoding the lysine decarboxylase into *Shigella flexneri *2a reduces its pathogenicity [8,9]. It thus appears that positive selection may act to remove the *cadA *gene from the genomes of *Shigella *strains. It is, however, hard to estimate the proportion of genes that have been lost due to positive selection. Unless there is information regarding the fitness effect of a specific gene loss event, it is not currently possible to distinguish whether it was lost adaptively or whether it was lost as the result of a reduction in purifying selection. To further complicate matters, cases of gene loss that are due to reduction in purifying selection may lead to other gene loss events that may be adaptive. For example, loss of flagellar genes may occur in *Shigella *due to a pathway-specific reduction in purifying selection, as *Shigella *strains do not need flagella for their motility. However, once the flagellar function is lost, it may be adaptive to lose the master activator of the flagellar pathway, as this will ensure that the remaining flagellar genes will not be expressed and energy will not be wasted [21,24]. In addition, once the flagellar function is no longer useful it may be adaptive to stop producing flagella completely due to the strong immunogenic properties of the flagellar apparatus.

It is important to note that in addition to reductions in the effectiveness of purifying selection, positive selection may also increase the average dN. It is very hard to distinguish between the two sources of increase in dN, as both may act together. It is possible to attempt to estimate whether significantly more adaptations are occurring in *Shigella *by considering genes that have clearly been subject to positive selection and have dN/dS values that are significantly higher than one. If indeed positive selection is much stronger in all *Shigella *strains compared to all *E. coli *strains, we may find more such genes in *Shigella *than in other pathogenic *E. coli *strains. Such an examination is far from conclusive as genes may be under positive selection even if the ratio between dN and dS is lower than one. Moreover, reduced effectiveness of purifying selection in *Shigella *may also increase the ratio of dN and dS, which together with similar levels of positive selection may lead to more genes in *Shigella *having dN/dS >> 1. Nevertheless, such an analysis may give some indication as to whether positive selection is more abundant for *Shigella*. Genes for which dN/dS is significantly higher than one are rare in both *E. coli *and *Shigella *and their numbers do not seem to differ much between *Shigella *and other pathogenic *E. coli*. The *Shigella *strain with the most genes with dN/dS larger than one (*Shigella flexneri *2a) has 11 such genes while the pathogenic *E. coli *strain with the most such genes (*E. coli *O157:H7 EDL 933) has 8. Only 1 to 4 genes in each of the examined *Shigella *strains have dN/dS values larger than 1.5. The same is true for the other pathogenic *E. coli *strains. While this indicates that not many more genes in *Shigella *are under very strong positive selection than in other pathogenic *E. coli*, it does not prove that positive selection does not affect dN more strongly in *Shigella*. However, our finding that the genes that are lost more readily are those that are less constrained in the organisms in which they are maintained points towards reduced purifying selection playing a prominent role in increasing gene loss.

A recent study [25] has attempted to test the rate of adaptive evolution in enteric bacteria by applying a methodology based on the McDonald-Kreitman test. The McDonald-Kreitman test estimates adaptation by comparing the ratio of non-synonymous to synonymous polymorphisms within a population to the same ratio in substitutions that occur between species [26]. Charlesworth *et al*. [25] examined six strains of *E. coli *and six strains of *Salmonella enterica *and considered each group of six strains to represent a separate species. They counted differences between different *E. coli *strains as polymorphisms and differences between an *E. coli *strain and a *Salmonella *strain as substitutions. Based on this they calculated the percentage of adaptive substitutions to be around 50%. The McDonald-Kreitman test and the methodologies that were derived from it all rely on certain assumptions. Among these is the assumption that the evolutionary process is stationary [26]. We demonstrate that this key assumption does not hold for all enteric bacteria, as we show that the effectiveness of purifying selection is different for different groups of enteric bacteria. For instance, if MacDonald-Kreitman tests utilize polymorphism levels in *Shigella *rather than in *E. coli*, they would yield different estimates for the percentage of adaptive substitutions. It may thus be important to use a variety of sources for polymorphism data to test for robustness of McDonald-Kreitman tests.

In this study we looked at two groups of very closely related pathogens that are, in fact, all clones of *E. coli*. Even among these two close groups we found significant differences in the rate of gene loss that correlate with differences in population structure, lifestyle and niche. It would be interesting to examine additional groups of pathogens to see whether there is a gradient in the rate of gene loss that correlates with pathogenic lifestyle. This should fine-tune and quantify the claim that pathogens lose genes at higher rates than free-living organisms and may shed additional light on the forces that determine the rates of gene loss and on its effect on pathogenicity.

## Conclusion

In this study we examined the rates of gene loss in a relatively large number of pathogenic *E. coli *and *Shigella *strains and correlated them with the genome-wide effectiveness of purifying selection. We demonstrate that gene loss is accelerated for *Shigella *compared to other pathogenic *E. coli*. Our results show that this observed acceleration in the rate of gene loss is attributable at least in part to a genome-wide reduction in purifying selection. This study demonstrates that purifying selection acts with different effectiveness in different facultative pathogens and that this difference affects the rate of gene loss.

## Materials and methods

### Determining the absence or presence of *E. coli *K12 genes in the other bacteria studied

Gene sequences for the 2,394 *E. coli *K12 genes were extracted from GenBank version NC_000913.1 of the *E. coli *K12 genome, and annotations of the genes were extracted from the Ecogene database [17]. The genomic sequences of *E. coli *O157:H7, *E. coli *O157:H7 EDL933, *E. coli *APEC 01, *E. coli *CFT073, *E. coli *536, *E. coli *UTI89, *Shigella flexneri *2a, *Shigella flexneri *2a 2457T, *Shigella flexneri *5 8401, *Shigella sonnei *Ss046, *Shigella boydii *Sb227, *Shigella dysenteriae *Sd197, and *Salmonella typhimurium *LT2 were downloaded from the NCBI ftp server [27]. As each genome project annotates the protein-coding genes within its genome using different methods and different thresholds, we did not wish to rely on the annotations provided with the genome sequences. Instead, we relied only on the more experimentally verified annotation of *E. coli *K12 genes provided in the Ecogene database [17] and developed a methodology for detecting gene absence in very closely related bacteria that does not rely on the annotation of the other genomes: Each *E. coli *K12 protein-coding gene was compared at the DNA level to the complete genomic sequence of each of the 13 other bacteria using a locally installed version of the FASTA program [28], and the best hit and percentage identity were recorded for each organism. If in a certain bacterium the best hit was conserved across less than 40% of the *E. coli *K12 gene sequence, the gene was marked as absent from that genome. If the best hit was conserved across 40% or more of the *E. coli *K12 gene sequence, the sequence corresponding to the *E. coli *K12 full length gene was extracted from the bacterium genomic sequence and translated. The translated sequence was then examined for the presence of stop codons. If a stop codon appeared in the sequence so that the final length of the translated protein was less than 80% of the *E. coli *K12 protein, the gene was marked as absent from the genome. Genes for which this was not the case were compared at the protein level to the *E. coli *K12 corresponding protein sequences. If the best hit of this comparison was conserved across less than 80% of the *E. coli *K12 protein sequence, the gene was marked as absent from the genome. Otherwise the gene was marked as present. It is important to note that the examined strains are all clones of *E. coli *and are thus extremely closely related to *E. coli *K12. Thus, the sequences of orthologous genes in these strains are highly similar. This makes the determination of a gene's absence much more straight forward than for more distantly related organisms.

### Pairwise substitution rate calculations

For each of the 2,394 genes in our dataset, we generated a pairwise alignment between the sequence of the *E. coli *K12 gene and the corresponding sequence in each of the pathogenic *E. coli *and *Shigella *strains in which this gene was conserved (based on the results of the process described in the previous section). Alignments were created using a locally installed version of the FASTA program [28]. Based on these alignments we calculated dN/dS for each sequence pair as described in Nei and Gojobori [29].

In order to study the rate of evolution of genes that are conserved in all the examined strains and of those that are conserved in only some of the strains, we followed the following strategy: first, a pairwise dN/dS of all the genes conserved in each of the 12 pathogenic bacteria was calculated against the *E. coli *K12 corresponding sequences. We then normalized these dN/dS values within each strain by calculating a Z-score:

$$Z(gene,strain)=\frac{dN/dS(gene,strain)-\bar{dN/dS}(strain)}{\sigma_{dN/dS}(strain)}$$

As a final step, the normalized values of each gene in each of the strains in which that gene was conserved were averaged across strains. This resulted in each gene receiving a single value that represents its rate of evolution in the bacteria in which it is present.

### Construction and comparison of phylogenetic trees

Of the 2,394 genes in our dataset, 1,214 are present in all the pathogenic *E. coli *and *Shigella *strains as well as in *S. typhimurium *and can be aligned across their entire sequence to the *E. coli *K12 sequence in all of these organisms. From these genes, we selected at random 100 genes. We concatenated the sequences of these genes and aligned them using the slow and accurate version of the ClustalW algorithm. We created 100 bootstrapping datasets from these alignments using the Phylip package Seqboot program [30]. These datasets were then used to construct 100 trees using the Phylip DNAml program [30], with *S. typhimurium *serving as an outgroup. The trees were then consolidated using the Phylip Consense program [30]. The tree representing the phylogeny of the *prpB *gene in the *E. coli *strains (Figure 4) was inferred using the same programs. In order to estimate the significance of the differences between trees we used the Shimodaira-Hasegawa test [20,31] as implemented in Tree-puzzle 5.2 [32].

### Calculating the average dN and dS along each branch of the tree

The sequences of each of the 1,214 genes in our dataset that are present in all the pathogenic *E. coli *and *Shigella *strains as well as in *S. typhimurium *were aligned separately using the slow and accurate version of the ClustalW algorithm. We ran the PAML Codeml program [18] on these alignments using the free-ratio model in order to estimate the rate of synonymous (dS) and non-synonymous (dN) mutations, for each gene, along each branch of the tree. Average dS and dN values were then calculated for each branch of the tree.

### Statistical analyses

We modeled the number of genes lost along a branch as a Poisson random variable, whose mean parameter may depend on dN, dS and whether the branch leads to *E. coli *or *Shigella *strains. We examined various hypotheses using Poisson regression models, which were fitted using the glm function in R. The two types of branches are coded by an indicator variable that is zero if the branch leads towards an *E. coli *strain and is one if the branch leads towards a *Shigella *strain.

To test whether gene loss occurs at different rates along the two types of branches adjusting for dS, we fitted the model:

*E*(*Y*) = *f*(*β*_0 _+ *β*_1_*dS *+ *β*_2_*I*_*shigella*_)

where Y denotes the number of genes lost, f is the Poisson link function [33], and I_*Shigella *_is a binary indicator variable denoting whether the branch leads to an *E. coli *strain (I_*Shigella *_= 0) or to a *Shigella *strain (I_*Shigella *_= 1). The one branch that leads to mixed strains was omitted from all analyses. Testing whether gene loss occurs at different rates along the two types of branches amounts to testing the null hypothesis β_2 _= 0.

To test whether dN adds information about gene loss beyond dS, we computed the likelihood ratio statistic for two nested Poisson regression models. The null model is similar to equation 1 except that we adjusted for dS alone, while the extended alternative model adjusts for both dS and dN. The log-likelihood of Y given dS (dS and dN for the extended model) can be derived from the residual deviance D(y; dS) = -2log P(Y | dS) (D(y; dS, dN) = -2log P(Y|dS, dN) for the extended model). Under the null hypothesis that dN does not capture more information about Y beyond dS, the likelihood ratio (or equivalently the reduction in deviance) follows a χ^2 ^with df = 1.

## Abbreviations

EHEC, enterohaemorrhagic *E. coli*.

## Authors' contributions

RH designed and performed the research and wrote the manuscript. HT provided statistical advice and performed statistical analyses. DP designed the research and co-wrote the manuscript.
